# Reliability assessment of CT enhancement rate and extracellular volume in liver fibrosis prediction

**DOI:** 10.1186/s12876-025-03678-5

**Published:** 2025-02-21

**Authors:** Faeze Salahshour, Aminreza Abkhoo, Sina Sadeghian, Masoomeh Safaei

**Affiliations:** 1 Advanced Diagnostic and Interventional Radiology Research Center (ADIR), Imam Khomeini Hospital, Tehran University of Medical Sciences, Tehran, Iran; 2 Department of Pathology, Cancer Institute, Imam Khomeini Hospital Complex, Tehran University of Medical Sciences, Tehran, Iran

**Keywords:** Liver fibrosis, Extracellular Space, Portal vein, Tomography, X-Ray computed, Contrast media, Predictive value of tests

## Abstract

**Background:**

Reliable, non-invasive evaluation of liver fibrosis is essential for early disease management. Computed tomography (CT)-based extracellular volume (ECV) fraction and portal venous phase enhancement rate (VP-ER) have shown potential in quantifying mild-to-moderate fibrosis. This study investigates the diagnostic performance of ECV and VP-ER in differentiating non-significant (F0–F1) from significant (F2–F3) fibrosis in biopsy-confirmed patients.

**Methods:**

Ninety-three patients (20–72 years, 56.9% male) undergoing liver biopsy and multiphasic CT scans were retrospectively enrolled. Patients with METAVIR F4 cirrhosis or incomplete imaging/pathological data were excluded. Hematocrit levels were obtained on the day of CT. ECV was calculated from differences in liver and aortic attenuation between delayed and enhanced phases, adjusted for hematocrit. VP-ER was derived as the ratio of liver attenuation in venous to portal venous phases multiplied by 100. Spearman’s correlation, receiver operating characteristic (ROC) curves, and DeLong tests evaluated their performance. Multiple logistic regression assessed independent contributions of ECV and VP-ER to fibrosis status.

**Results:**

Fifty-three patients had no significant fibrosis (F0–F1) and 40 had significant fibrosis (F2–F3). ECV demonstrated a moderate correlation with fibrosis grade (*r* = 0.531, *p* < 0.0001), while VP-ER showed a weaker yet statistically significant correlation (*r* = 0.363, *p* = 0.0003). ROC analyses yielded an area under the curve (AUC) of 0.698 for ECV (cut-off = 38%) and 0.763 for VP-ER (cut-off = 71%), with no significant difference between AUCs (*p* = 0.358). VP-ER accurately classified 70 patients, while ECV correctly predicted 65. Logistic regression revealed significant associations for both VP-ER (OR = 1.08; *p* = 0.007) and ECV (OR = 1.025; *p* = 0.0132), achieving 72.04% classification accuracy and an overall AUC of 0.756 (95% CI: 0.688–0.863).

**Conclusion:**

ECV fraction and VP-ER demonstrated reliable, complementary capabilities for distinguishing non-significant fibrosis from significant fibrosis. Their combined use in routine multiphasic CT protocols may reduce dependence on invasive biopsy while offering robust sensitivity and specificity for early fibrosis assessment. Further studies including cirrhotic populations and larger cohorts are recommended.

## Introduction

Liver fibrosis is a critical determinant of patient prognosis in chronic liver diseases, progressing from mild fibrosis (F0-F1) to cirrhosis (F4) [1]. Accurate staging is essential for diagnosing, managing, and treating liver diseases, as it guides therapeutic decisions and predicts outcomes [2]. Traditionally, liver biopsy has been the reference standard for fibrosis assessment; however, it is invasive, subject to sampling errors, and carries potential complications [3, 4]. Consequently, there is a growing need for reliable non-invasive biomarkers to evaluate liver fibrosis effectively [5–7].

Current non-invasive approaches include magnetic resonance imaging (MRI), ultrasound elastography, and serum biomarkers. While these methods offer advantages over biopsy, they may sometimes be unavailable or impractical, particularly in asymptomatic patients or settings lacking necessary resources [5–7]. Therefore, the introduction of new methodologies for effective liver fibrosis assessment remains essential [8, 9].

Computed tomography (CT) presents a non-invasive modality to assess liver tissue characteristics. Among CT-based metrics, the extracellular volume (ECV) fraction and portal venous phase enhancement rate (VP-ER) have emerged as promising indicators of fibrotic burden. The liver’s complex architecture comprises several compartments, including the intercellular space, extracellular extravascular space (EES), and intravascular space [10]. ECV quantifies the expansion of the extravascular extracellular space, primarily due to extracellular matrix (ECM) deposition, such as collagen accumulation, which is a hallmark of fibrosis progression [11, 12]. ECV is calculated as the fractional extracellular space (fEES) plus the intravascular space, measured through contrast-enhanced CT scans using water-soluble, low molecular weight (≤ 200 Da) contrast agents that equilibrate between these compartments [13–15].

The ECV fraction was first employed to determine the fibrosis grade in the human liver [16]. Despite its high effectiveness in fibrosis diagnosis, earlier studies reported delayed imaging times ranging from 180 to 600 s, which poses practical challenges for routine clinical implementation [16]. Moreover, CT imaging alone does not suffice for fibrosis diagnosis, necessitating comprehensive assessment through multiphasic scanning protocols.

Liver enhancement patterns, particularly in the portal venous phase, have been correlated with fibrosis severity. The portal venous phase enhancement rate (VP-ER) quantifies the parenchymal enhancement ratio between the portal venous and hepatic venous phases, reflecting alterations in hepatic perfusion and vascular resistance associated with fibrosis progression [17, 18]. Research indicates that both ECV and VP-ER serve as non-invasive indicators of fibrosis, with VP-ER demonstrating a direct correlation with fibrosis in patients with potential cirrhosis [17] and ECV being recognized as an efficient liver fibrosis marker [16]. However, previous studies were limited by small sample sizes and the lack of pathological fibrosis confirmation [16, 17].

In evaluating chronic liver disease, early and accurate identification of significant fibrosis (F2–F3) is critical for guiding patient management. CT-based parameters, including ECV fraction and VP-ER, have shown promise in detecting and staging hepatic fibrosis. Despite their potential, the reliability and diagnostic performance of ECV and VP-ER in predicting liver fibrosis stages, particularly in non-cirrhotic populations, remain underexplored. Previous studies have demonstrated correlations between these CT metrics and fibrosis severity, but variations in imaging protocols and patient populations necessitate further validation [19].

Although cirrhosis (F4) reflects a pivotal endpoint of hepatic fibrosis, the advanced structural remodeling at this stage [20] may confound quantitative imaging signals like ECV and VP-ER. By restricting our cohort to F0–F3, we aimed to isolate the specific contrast-uptake characteristics associated with no significant to severe fibrosis, enhancing the interpretability of our findings. Furthermore, the fractional extravascular extracellular space (fEES) expands with fibrosis as excess collagen accumulates in the liver parenchyma [21], yet standardizing a direct fEES measure remains technically challenging. Hence, we leverage ECV—a validated surrogate that quantifies the distribution of extracellular, low-molecular-weight contrast agents—thereby providing a stable index of matrix expansion [17].

Despite these encouraging data, the diagnostic performance of ECV and VP-ER requires further validation. Excluding advanced cirrhosis (F4) helps isolate fibrotic changes that precede substantial architectural distortion. Accordingly, the present study leverages the histopathological reference standard to evaluate the relationship of CT-derived ECV and VP-ER with liver fibrosis grades in a biopsy-confirmed population. Through this work, we aim to (i) determine each parameter’s diagnostic utility for distinguishing non-significant fibrosis (F0–F1) from significant fibrosis (F2–F3), (ii) explore their reproducibility via inter-observer reliability, and (iii) investigate whether combining ECV and VP-ER enhances fibrosis detection relative to individual metrics. By focusing on F0–F3 cases, we seek to characterize the core fibrotic changes in a manner translatable to clinical settings, potentially introducing more reliable non-invasive methods for early fibrosis assessment.

## Materials and methods

### Study design

The Institutional Review Board (IRB) of Tehran University of Medical Sciences (Ethics code: IR.TUMS.IKHC.REC.1402.035) reviewed and approved this retrospective, cross-sectional study in compliance with the Declaration of Helsinki. The IRB also confirmed that the requirement for informed consent was waived, given the retrospective nature of the project and the use of anonymized patient records. The study included patients who underwent liver biopsy procedures, either percutaneous or surgically, at a tertiary care hospital between January 2013 and December 2022. Indications for liver biopsy included liver transplantation, elevated liver enzyme levels, and suspicious hepatic masses. Patients classified as METAVIR F4 (cirrhosis) were excluded from the study to focus on assessing no significant to severe fibrosis stages (F0–F3).

A power analysis was conducted using G*Power (version 3.1.9) to determine the required sample size. It was estimated that a sample size of approximately 90 would provide at least 80% power (α = 0.05) to detect moderate effect sizes in correlations (Spearman’s rho ≥ 0.3) between imaging metrics and fibrosis stages.

### Patient selection

Inclusion criteria were the availability of complete imaging data, including unenhanced, portal venous, and delayed phases, and hematocrit levels measured on the day of the CT examination. Exclusion criteria encompassed patients with incomplete imaging or pathological data, fibrosis grading performed by systems other than METAVIR, and contrast-enhanced CT scans conducted outside the 1-month window before or after liver biopsy. Additionally, patients with METAVIR F4 fibrosis were excluded. Out of an initial 112 patients screened, 19 were excluded due to incomplete imaging (12 patients) and classification as F4 (7 patients). The final sample comprised 93 patients.

### Imaging acquisition

CT scans were performed using a Lightspeed 64-detector CT (Sixteen Silences, Erlangen, Germany) and a Siemens Somatom Emotion MDCT scanner (GE Healthcare, Milwaukee, USA). A non-ionic contrast agent (Omnipaque, Daiichi Sankyo, Tokyo, Japan) was administered intravenously at a rate of 3 ml/s, followed by a 30 ml saline flush. The total contrast volume was calculated as 1 ml/kg body weight. Scan parameters included a tube voltage of 120 kV, tube current of 600 mA, rotation time of 0.5 s, and collimation of 0.625 mm. Reconstruction slice thickness ranged from 0.625 to 2.5 mm. CT images were acquired in the unenhanced phase, portal venous phase (PVP) at 60–70 s post-injection, and venous phase (VP) at 180 s post-injection.

### Image interpretation

**Fig. 1:**
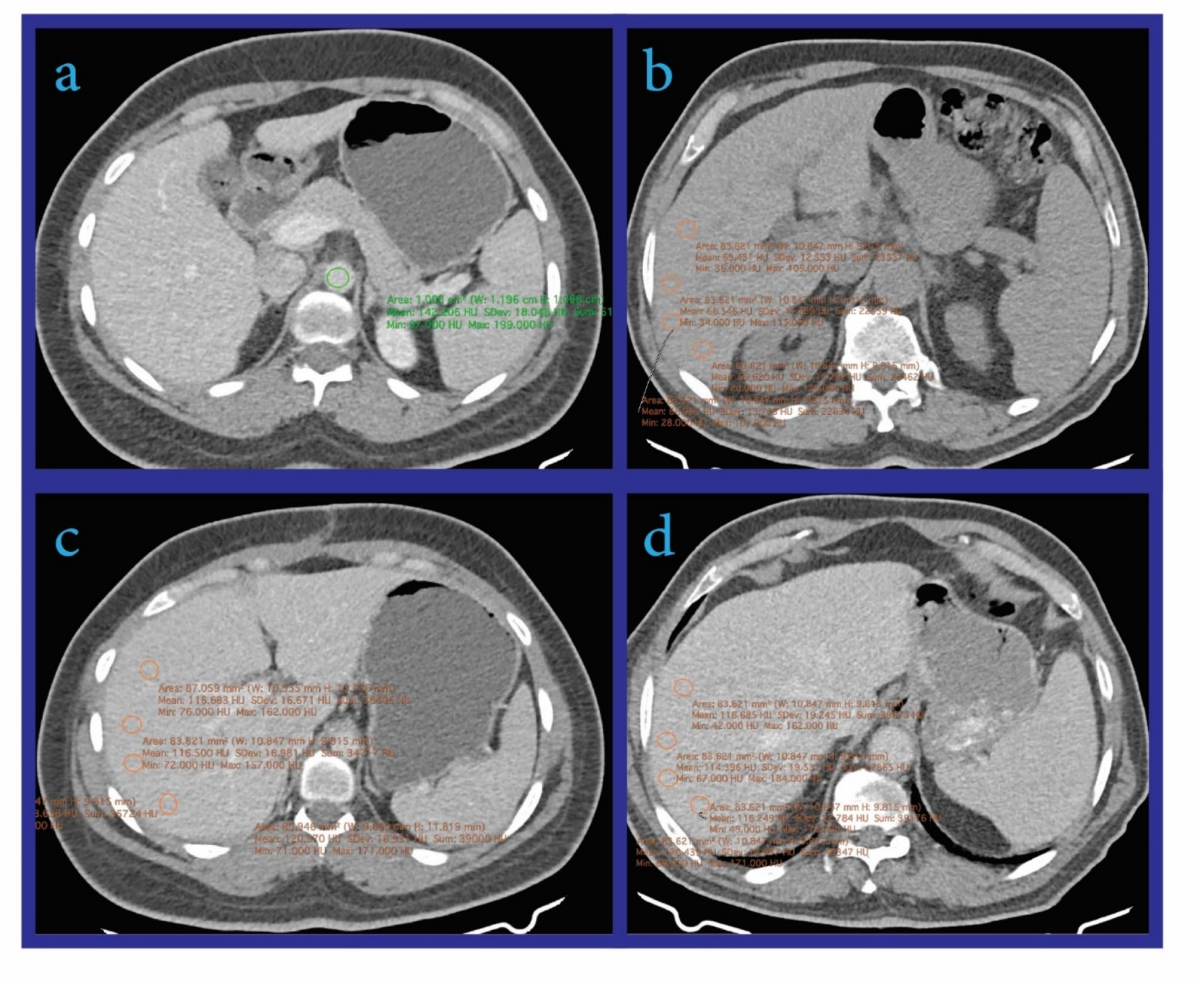
Schematic Diagram of ROI Placement. A four-panel image showing CT slices with elliptical ROI placed in the abdominal aorta, avoiding atheromatous plaques **(a)**, and multiple circular ROIs (10 mm) placed in the liver parenchyma: **(b)** Unenhanced phase, **(c)** Portal Venous Phase (PVP) at 60–70 s, and **(d)** Venous Phase (VP) at 180 s

CT images were evaluated by an abdominal radiologist with seven years of experience using the INFINITT image archiving and communication system (INFINITT Healthcare, Seoul, South Korea). Regions of interest (ROIs) were placed as illustrated in Fig. 1. Circular ROIs with 10-mm diameters were positioned in the liver parenchyma (four in the right lobe and three in the left lobe) across the unenhanced, PVP, and VP phases. An elliptical ROI was placed in the abdominal aorta, avoiding atheromatous plaques and the aortic wall. Large blood vessels, suspicious parenchymal lesions, bile ducts, the liver capsule, and the diaphragm were excluded from the ROIs (See Fig. 1). Mean Hounsfield Units (HU) were recorded for each ROI, and the VP-ER was calculated using the formula:

$$\:VP-ER=\left(\frac{{HU}_{VP}}{{HU}_{PVP}}\right)\times\:100$$

Hematocrit (HCT) levels were measured on the day of each CT scan, and the extracellular volume (ECV) fraction was calculated as:

$$\:ECV = (1 - HCT) \times \:\left( {\frac{{\Delta \:H{U_{liver}}}}{{\Delta \:H{U_{Aorta}}}}} \right)$$

where ΔHU represents the HU difference between the delayed phase and enhanced images.

### Pathological grading

Liver biopsy specimens were obtained either surgically or percutaneously for patients requiring liver transplantation. Two pathologists with fifteen and eight years of experiences, blinded to imaging and clinical data, reviewed the specimens using the METAVIR scoring system. The METAVIR scoring system classifies fibrosis into: F0–F1 (no or mild fibrosis, no septa), F2 (moderate fibrosis, few septa), F3 (severe fibrosis, many septa but no cirrhosis), and F4 (cirrhosis).”

### Data analysis

Data normality was assessed using the Shapiro-Wilk test, which indicated a non-normal distribution. Spearman’s correlation was utilized to evaluate the relationships between ECV fraction, VP-ER, and fibrosis grades (F0-3). Correlation coefficients in the ranges of 0.2–0.4, 0.4–0.7, and 0.7–0.9 reflect weak, moderate, and strong correlations, respectively. Receiver Operating Characteristic (ROC) curves were generated to assess the diagnostic performance of ECV fraction and VP-ER, with the Area Under the Curve (AUC) serving as the performance metric. The Delong test was employed to compare the AUCs of ECV and VP-ER. Optimal cut-off values were determined using the Youden index to maximize specificity and sensitivity. Potential confounders, such as comorbidities and medication history, were documented and controlled for in the analysis. No significant differences in major comorbidities were observed between fibrosis groups.

To evaluate measurement consistency, intra- and inter-observer reliability analyses were conducted. For inter-observer reliability, two board-certified abdominal radiologists (each with ≥ 5 years of experience) independently measured ECV and VP-ER in a random subset of 30 patients. Intraclass correlation coefficients (ICCs) were computed using a two-way random-effects model with absolute agreement. Test-retest reliability was assessed in 20 patients who underwent a second CT scan within a two-week interval under identical imaging conditions.

Multiple logistic regression analysis was conducted to quantify the independent contributions of ECV and VP-ER to fibrosis diagnosis. The model included ECV and VP-ER as independent variables, with fibrosis status (significant vs. no significant) as the dependent variable. Odds Ratios (OR) and 95% Confidence Intervals (CI) were calculated to evaluate the significance of the predictors. The accuracy of the model was assessed, and the ROC curve of the predicted probabilities was generated.

## Results

The study included **93 patients** aged between **20 and 72 years** (mean age = **47.7 ± 12.7 years**), with **56.9% male**. Out of the initial 112 patients, **19 were excluded** due to incomplete imaging (12 patients) and classification as F4 (7 patients). The final sample comprised **53 patients** with **No Significant Fibrosis (F0–F1)** and **40 patients** with **Significant Fibrosis (F2–F3)**. The baseline characteristics and quantitative imaging data of the patients are summarized in Table 1.

**Table 1:** Patient characteristics and quantitative Image Data

Characteristic	Total (*N* = 93)	No Significant Fibrosis (F0–F1, *N* = 53)	Significant Fibrosis (F2–F3, *N* = 40)	*P*-value
Sex (Male)	53 (56.9%)	26 (49.1%)	27 (67.5%)	0.742
Age (years)	47.7 ± 12.7	47.8 ± 14.2	47.8 ± 11.2	0.925
Hematocrit (%)	34.4 ± 4.3	35.7 ± 4.3	33.2 ± 3.9	0.005
ECV (%)	37.4 ± 7.7	34.9 ± 7.1	39.9 ± 7.5	0.002
VP-ER (%)	69.5 ± 35.5	57.8 ± 20.1	81.6 ± 43.3	< 0.001
BMI (kg/m²)	27.1 ± 3.9	26.5 ± 4.0	27.9 ± 3.8	0.078
Major Comorbidities*	12 (12.9%)	7 (13.2%)	5 (12.5%)	0.865

* *N* (%), all other variables reported as Mean (standard deviation). Mann-Whitney test and chi-squared were used for between-group comparisons. ECV: extracellular volume, VP-ER: portal venous phase enhancement rate. *BMI was reported for completeness. “Major Comorbidities” include chronic conditions such as diabetes, rheumatologic diseases, and hypertension

Reliability analyses demonstrated excellent reproducibility for both ECV and VP-ER. The test-retest ICCs were 0.86 for ECV and 0.84 for VP-ER, while inter-observer and Intra-Observer ICCs were 0.88 and 0.89 for ECV, and 0.85 and 0.83 for VP-ER (see Table 2).

**Table 2:** Reliability of ECV and VP-ER measurements

Parameter	Test-Retest ICC (95% CI)	Inter-Observer ICC (95% CI)	Intra-Observer ICC (95% CI)
ECV	0.86 (0.78–0.92)	0.88 (0.82–0.93)	0.89 (0.81–0.95)
VP-ER	0.84 (0.75–0.89)	0.85 (0.78–0.90)	0.83 (0.74–0.89)

Intra-class correlation coefficients (ICCs) demonstrating test-retest, inter-observer and Intra-Observer reliability for ECV and VP-ER measurements

**Fig. 2:**
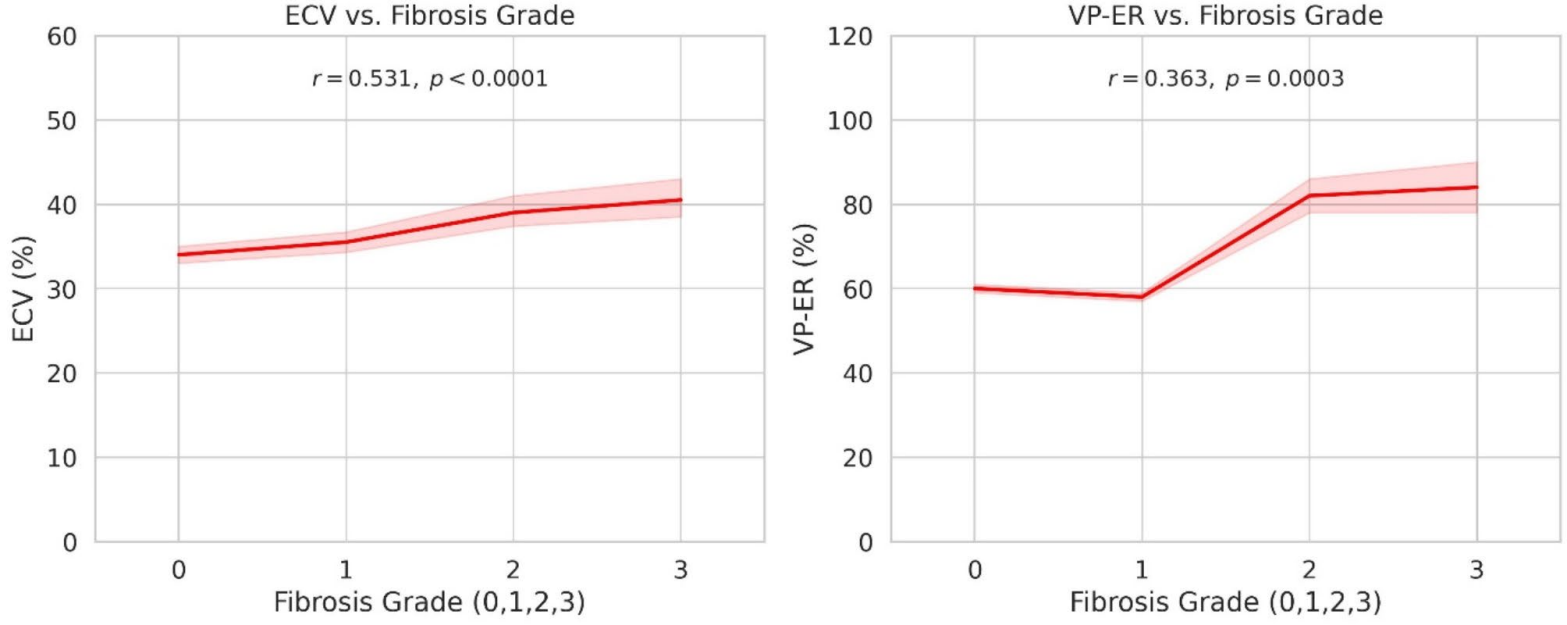
Spearman’s Correlation between CT Parameters and Liver Fibrosis Grades

Spearman’s correlation (Fig. 2) indicated a moderately positive relationship between ECV and fibrosis grade (*r* = 0.531, *p* < 0.0001), whereas VP-ER displayed a somewhat weaker but still significant correlation (*r* = 0.363, *p* = 0.0003).

**Fig. 3:**
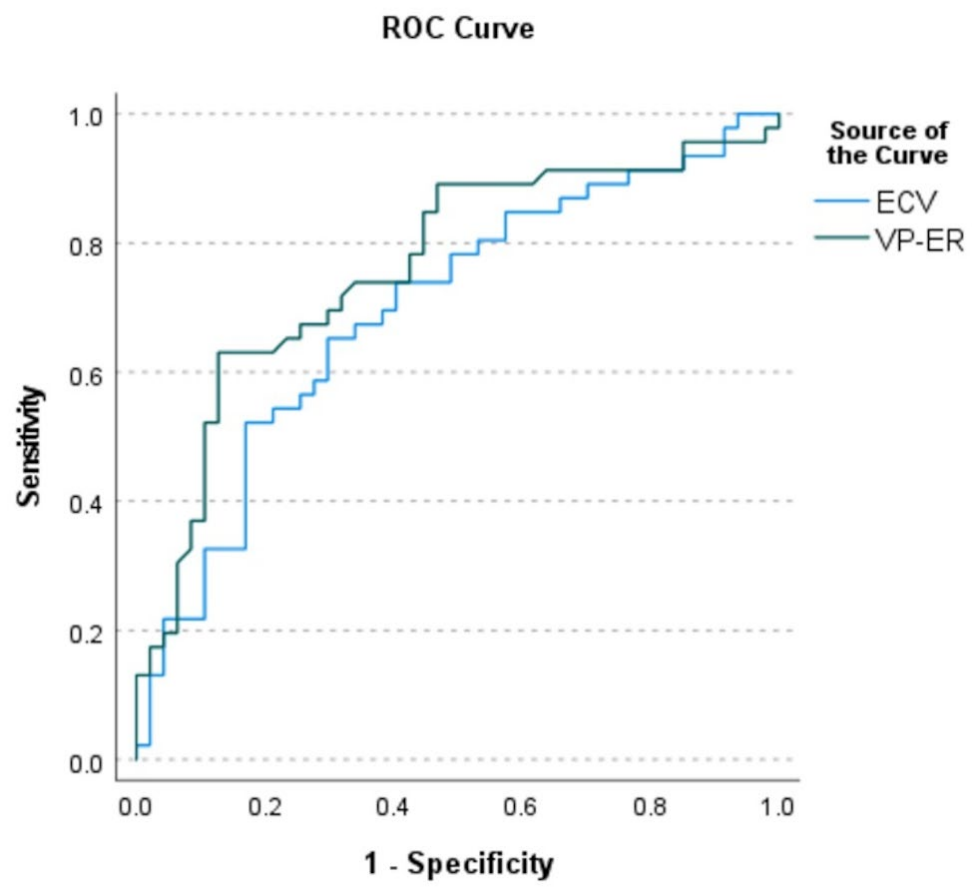
Receiver operating characteristic (ROC) Curves for ECV and VP-ER in detecting fibrosis. Displays the ROC curves with 95% confidence intervals and optimal cut-off points for VP-ER and ECV

**ROC curves** were utilized to assess the diagnostic performance of ECV and VP-ER in detecting fibrosis. Figure 3 presents the ROC curves, illustrating the AUCs for VP-ER and ECV.

The AUC for VP-ER was **0.763** (95% CI: 0.664–0.845) and for ECV was **0.698** (95% CI: 0.594–0.788). The Delong test indicated no significant difference between the AUCs of VP-ER and ECV (*p* = 0.358). The optimal cut-off values were determined to be **71%** for VP-ER (Youden’s J = 0.50, *p* < 0.0001) and **38%** for ECV (Youden’s J = 0.35, *p* = 0.0003). At these cut-offs, VP-ER correctly classified **70** patients, while ECV accurately predicted **65** cases.

**Multiple logistic regression analysis** demonstrated that both ECV and VP-ER significantly predicted pathological liver fibrosis. The results are detailed in Table 3.

**Table 3:** Multiple regression analysis Predicting Pathological Liver Fibrosis

PREDICTOR	Odds Ratio	95% CI	*P*-value
VP-ER	1.08	1.01–1.16	0.007
ECV	1.025	1.00-1.04	0.0132

ECV: Extracellular volume; VP-ER: Portal venous phase enhancement rate

The logistic regression model achieved an accuracy of **72.04%** and an ROC AUC of **0.756** (95% CI: 0.688–0.863, *p* < 0.0001). Multiple regression indicated that each 1-unit increase in VP-ER was associated with an 8% increase in the odds of significant fibrosis (OR = 1.08, *p* = 0.007), whereas ECV conferred a 2.5% odds elevation per unit rise (OR = 1.025, *p* = 0.0132).

## Discussion

The present study substantiates the efficacy of ECV fraction and VP-ER in differentiating non-significant fibrosis (F0–F1) from significant fibrosis (F2–F3) in non-cirrhotic patients. Our findings demonstrate clear trends of both ECV and VP-ER increasing with fibrosis stage, significant correlations with histologically determined fibrosis, and satisfactory diagnostic performance as reflected by area under the ROC curve (AUC) values. Multiple logistic regression further underscores their combined utility in predicting fibrosis status with a classification accuracy of 72.04%. These observations corroborate existing literature that identifies ECV and VP-ER as viable non-invasive imaging biomarkers for hepatic fibrosis.

Our ECV fraction showed a moderate correlation with fibrosis stage (Spearman’s rho = 0.531) and an AUC of 0.698. These findings align with earlier studies highlighting ECV as a surrogate for collagen deposition within the extracellular matrix. In particular, prior investigations have reported comparable AUCs ranging from 0.62 to 0.82 for fibrosis detection using ECV measurements [17, 22, 23]. For instance, Yoon et al. described an AUC of 0.832 for identifying significant fibrosis (F ≥ 2), albeit with a lower ECV threshold of 28.76% [22]. The slight differences in AUC values and optimal cut-offs across studies may be attributed to variations in imaging protocols, contrast timing, and patient demographics. Additionally, Liang et al. showed that ECV correlates strongly with serum markers of fibrogenesis, indicating that it captures the biological progression of the extracellular matrix [24].

We found VP-ER to be a robust discriminator of significant fibrosis, with an AUC of 0.763, which is comparable to the AUC of 0.75 reported in a larger cohort by Masuda et al. [17]. Their correlation analysis (*r* = 0.37) also matches our observation that VP-ER reflects hemodynamic alterations occurring alongside fibrotic tissue remodeling. The observed consistency underscores VP-ER’s potential as a practical quantitative biomarker, possibly outperforming ECV in some populations where vascular flow changes become prominent earlier in fibrosis progression [17].

An elevated ECV fraction in fibrotic livers is primarily attributed to the accumulation of extracellular matrix components, including collagen, which expand the space into which contrast material distributes [25, 26]. As collagen deposition progresses, the delayed-phase concentration of contrast increases, producing higher ECV measurements. Our data are consistent with these mechanistic interpretations, supporting ECV’s role as a tissue-level index for fibrosis severity.

VP-ER reflects alterations in portal venous perfusion pathways, which become increasingly distorted as fibrosis advances. Rising vascular resistance and compromised portal flow in fibrotic livers contribute to changes in enhancement kinetics during the venous phase, facilitating the quantification of fibrosis-related perfusion shifts. This pathophysiologic link explains the good correlation observed in our study and others [17].

The combination of ECV and VP-ER leverages both structural (matrix expansion) and hemodynamic (perfusion alteration) indicators, providing a more comprehensive view of fibrotic changes than either marker alone. Our multivariable logistic regression suggests that each parameter exerts an independent contribution, highlighting the importance of including both in imaging-based fibrosis assessments. High intra- and inter-observer intraclass correlation coefficients (ICCs > 0.80) for both ECV and VP-ER confirm their reproducibility using standardized acquisition protocols. These metrics can be derived from routine multiphasic CT scans without extra contrast administration, supporting their feasibility for integration into standard clinical workflows [27]. Furthermore, the stable test-retest performance of these measures paves the way for longitudinal fibrosis monitoring, a critical aspect in chronic liver disease management.

Our findings extend the growing body of evidence supporting CT-based fibrosis quantification. Although MRI elastography (MRE) and ultrasound elastography are established non-invasive techniques, CT-derived measurements such as ECV and VP-ER offer unique benefits, including widespread availability and the potential for concurrent evaluation of other abdominal pathologies [23, 26]. As non-invasive imaging garners increasing interest for tracking disease progression, these data contribute to a more nuanced understanding of the respective strengths and limitations of various modalities.

While some investigators have focused on advanced fibrosis and cirrhosis [23, 27], our exclusive analysis of no significant to severe fibrosis (F0–F3) helps elucidate changes in contrast kinetics and extracellular matrix volume before end-stage cirrhotic transformations predominate. This approach yields a clearer signal-to-noise ratio in correlational analyses, minimizing confounding hemodynamic factors associated with portal hypertension and other sequelae of cirrhosis [28]. Comparative studies with MRI-based T1 mapping, MRE, and quantitative ultrasound would further clarify how CT-based biomarkers stack up against established techniques [26, 29].

Despite promising results, our study is constrained by several factors. The absence of F4 patients may limit applicability to cirrhotic populations, where advanced vascular remodeling could differentially affect ECV and VP-ER metrics. Additionally, our single-center, retrospective design with a sample size of 93 individuals restricts broader generalizability. As in other investigations, selection bias might also arise from the referral patterns at a tertiary healthcare facility, which typically manages complex liver disease cases [17, 22]. Although liver biopsy is considered the gold standard for fibrosis assessment, it is not without limitations. Sampling error remains a significant concern, as biopsies typically represent only 1/50,000th of the liver’s total mass, potentially leading to misclassification of fibrosis stage. Variability between pathologists in interpreting histological findings further compounds this issue, as fibrosis grading can be subjective and influenced by individual expertise. These factors may affect the accuracy of comparisons between imaging biomarkers and biopsy findings, underscoring the need for reliable non-invasive alternatives like VP-ER and ECV, which offer whole-liver assessment without the risks of sampling bias.

## Conclusions

ECV fraction and VP-ER emerge as robust, reproducible, and clinically actionable parameters for distinguishing non-significant fibrosis (F0–F1) from significant fibrosis (F2–F3) in a non-invasive manner. Their complementary pathophysiologic underpinnings—encompassing both extracellular matrix expansion and alterations in portal venous flow—underscore their synergistic value when employed jointly. By integrating ECV and VP-ER into routine multiphasic CT protocols, clinicians may enhance early fibrosis detection and monitoring, reduce reliance on invasive liver biopsy, and more accurately characterize disease progression. Future work addressing advanced fibrosis stages, broader patient cohorts, and cross-modality comparisons will further consolidate the role of ECV and VP-ER as pivotal biomarkers in chronic liver disease management.

## Data Availability

The datasets generated and/or analyzed during the current study are available from the corresponding author on reasonable request.

## Abbreviations

AUC: Area Under the Curve
CI: Confidence Interval
CT: Computed Tomography
ECM: Extracellular Matrix
ECV: Extracellular Volume
EES: Extravascular Extracellular Space
fEES: Fractional Extravascular Extracellular Space
HCT: Hematocrit
HU: Hounsfield Unit
MDCT: Multi-Detector Computed Tomography
METAVIR: Meta-analysis of Histological Data in Viral Hepatitis (fibrosis scoring system)
MRI: Magnetic Resonance Imaging
NPV: Negative Predictive Value
OR: Odds Ratio
PPV: Positive Predictive Value
PVP: Portal Venous Phase
ROC: Receiver Operating Characteristic
ROI: Region of Interest
VP: Venous Phase
VP-ER: Portal Venous Phase Enhancement Rate
