# Correction: Reliability assessment of CT enhancement rate and extracellular volume in liver fibrosis prediction

**DOI:** 10.1186/s12876-025-03741-1

**Published:** 2025-03-04

**Authors:** Faeze Salahshour, Aminreza Abkhoo, Sina Sadeghian, Masoomeh Safaei

**Affiliations:** 1 Advanced Diagnostic and Interventional Radiology Research Center (ADIR), Imam Khomeini Hospital, Tehran University of Medical Sciences, Tehran, Iran; 2 Department of Pathology, Cancer Institute, Imam Khomeini Hospital Complex, Tehran University of Medical Sciences, Tehran, Iran

**Correction**: ***BMC Gastroenterol* 25, 101 (2025)**

10.1186/s12876-025-03678-5

Following publication of the original article it was reported that there was an error in the authorship list. There was a spelling mistake in the name of Sina Sadeghian.

The wrong name was: Sina Sadeghia.

The correct name is: Sina Sadeghian.

The original article has been updated.

